# Targeting Parents for Childhood Weight Management: Development of a Theory-Driven and User-Centered Healthy Eating App

**DOI:** 10.2196/mhealth.3857

**Published:** 2015-06-18

**Authors:** Kristina Elizabeth Curtis, Sudakshina Lahiri, Katherine Elizabeth Brown

**Affiliations:** ^1^ The Institute of Digital Healthcare Warwick Manufacturing Group The University of Warwick Coventry United Kingdom; ^2^ Centre for Technology Enabled Health Research (CTEHR) Coventry University Coventry United Kingdom

**Keywords:** child, obesity, health behaviour, mHealth, healthy eating, evidenc-based, theory

## Abstract

**Background:**

The proliferation of health promotion apps along with mobile phones' array of features supporting health behavior change offers a new and innovative approach to childhood weight management. However, despite the critical role parents play in children’s weight related behaviors, few industry-led apps aimed at childhood weight management target parents. Furthermore, industry-led apps have been shown to lack a basis in behavior change theory and evidence. Equally important remains the issue of how to maximize users’ engagement with mobile health (mHealth) interventions where there is growing consensus that inputs from the commercial app industry and the target population should be an integral part of the development process.

**Objective:**

The aim of this study is to systematically design and develop a theory and evidence-driven, user-centered healthy eating app targeting parents for childhood weight management, and clearly document this for the research and app development community.

**Methods:**

The Behavior Change Wheel (BCW) framework, a theoretically-based approach for intervention development, along with a user-centered design (UCD) philosophy and collaboration with the commercial app industry, guided the development process. Current evidence, along with a series of 9 focus groups (total of 46 participants) comprised of family weight management case workers, parents with overweight and healthy weight children aged 5-11 years, and consultation with experts, provided data to inform the app development. Thematic analysis of focus groups helped to extract information related to relevant theoretical, user-centered, and technological components to underpin the design and development of the app.

**Results:**

Inputs from parents and experts working in the area of childhood weight management helped to identify the main target behavior: to help parents provide appropriate food portion sizes for their children. To achieve this target behavior, the behavioral diagnosis revealed the need for eliciting change in parents’ capability, motivation, and opportunity in 10-associated Theoretical Domains Framework (TDF) domains. Of the 9 possible intervention functions, 6 were selected to bring about this change which guided the selection of 21 behavior change techniques. Parents’ preferences for healthy eating app features revolved around four main themes (app features, time saving and convenience, aesthetics, and gamification) whereupon a criterion was applied to guide the selection on which preferences should be integrated into the design of the app. Collaboration with the app company helped to build on users’ preferences for elements of gamification such as points, quizzes, and levels to optimize user engagement. Feedback from parents on interactive mock-ups helped to inform the final development of the prototype app.

**Conclusions:**

Here, we fully explicate a systematic approach applied in the development of a family-oriented, healthy eating health promotion app grounded in theory and evidence, and balanced with users’ preferences to help maximize its engagement with the target population.

## Introduction

### Background

Within the field of mobile health (mHealth), seen as mobile devices such as mobile phones, personal digital assistants (PDAs), and other wireless devices supporting a medical or public health practice [1], it is the advent of the mobile phone accompanied by an explosion of commercial mHealth apps that has gained the most attention [2]. Health promotion apps are by far the most commonly downloaded mHealth apps [3] and aim to support users to start or reinforce one or more health behaviors (eg, nutrition apps) and/or reduce risk behaviors (eg, smoking cessation apps) [4]. To date, nutrition and diet apps represent the fastest growing area of health promotion apps [2]. It is now well documented that mobile phones offer a number of attributes that maximize their potential for supporting health behavior change interventions including their accessibility (eg, global proliferation, widespread adoption across socioeconomic and demographic populations, and ubiquity) [2], personal nature (eg, always on the person, emotional attachment, and connectivity) [5], and programming flexibility (eg, information tailoring, context aware capabilities, and automated sensors) [6,7]. Additionally, mobile phones offer benefits for researchers regarding implementation (eg, low cost, rapid scalability, ease of use, zero-geography, and low participant burden) and real-time monitoring, data collection, and analysis [1,8].

### The Potential for Mobile Health Apps in Childhood Weight Management

Mobile health (mHealth) tools are also particularly suitable when it comes to supporting parental involvement in childhood weight management interventions, where there is growing consensus among researchers and practitioners that novel approaches using the internet [9-11] and mHealth apps [8,12] should be explored. For example, their zero-geography feature means that access to apps is not restricted to locations and can be delivered directly to families in the comfort and privacy of their own home [8]. This is especially advantageous for a parent population that report lack of time, scheduling conflicts, and location difficulties as major barriers to attending childhood weight management programs [13]. Another benefit of mHealth apps is their ‘glanceable displays’ that can provide parents with a quick and coherent overview of their child’s health information, potentially increasing their engagement with children’s weight-related behaviors [5]. In addition, participants can continue to access an intervention long after completion, which is important in weight management where there are high rates of relapse [8].

Behavior change techniques (BCTs) are seen as the observable, replicable, and active ingredients in an intervention that directly bring about behavior change [14]. Certain BCTs, such as self-monitoring which have been shown to be effective for adult and childhood weight management [15-17], are optimized through this medium and continue to increase in their sophistication [7]. For example, in cases where parents report difficulties in monitoring children’s dietary behaviors [18], mobile phone features such as cameras can be employed for children’s dietary monitoring where parents and children can take pictures of their food [19]. This has been shown to be especially effective for helping users monitor and reflect on their eating and exercise behaviors [20]. Moreover, mHealth apps may offer a more detailed and accurate measure of dietary behaviors thus increasing the robustness of childhood weight management interventions where current studies are limited by self-report measures [7]. Additional techniques, such as role modeling behaviors, can also be effectively implemented through the use of games and health challenges that families can play together, allowing parents’ behaviors to influence their children’s behaviors [8].

### Approaches to Mobile Health Development

With regards to mHealth app development, there is growing consensus that mHealth interventions should be based on evidence, behavior change theory, and formative research with the target audience [21]. Despite this, several reviews of commercial health promotion apps have revealed a significant lack of evidence-based guidelines [22-24] and health behavior change theory [25,26] in their development processes. With regards to childhood weight management, results from a recent review involving 57 pediatric weight management apps indicated that an overwhelming majority of the apps (61%) did not use any recommended strategies or behavioral targets. Moreover, few apps targeted parents/families [12], a vital element when managing children’s weight [9,10,27,28]. However, evidence implies that mHealth apps with more evidence-based strategies are least popular amongst consumers [29]; suggesting commercial mHealth apps may be more engaging for consumers, despite their lack of theoretical content. Arguably, mHealth development would benefit from greater collaboration between experts in behavior change and the commercial app industry to help address these gaps [4,26].

In addition to theory, evidence, and engaging design principles, mHealth interventions should also have social validity with regards to acceptability amongst its stakeholders [30]. Consequently, there is a growing trend towards adopting a user-centered design (UCD) approach [31-33]. This is especially pertinent in the case of apps where approximately 26% of all apps downloaded are discarded after first use [34].

### Theoretical Framework Guiding the Study

While theories and models of behavior change (eg, theory of planned behavior and transtheoretical model [35,36]) help guide intervention designers on which theoretical constructs to target in an intervention to elicit behavioral change, intervention development frameworks (eg, intervention mapping and/or Medical Research Council framework [37,38]) provide guidance on the development of a "coordinated set of activities" to help translate theory into practice [39]. However, the majority of the prominent theories and models of behavior change fail to take into account the context in which a behavior occurs, fail to focus on reflective processes (eg, attitudes and intentions), are static in structure, and are unable to explicitly state how to bring about change [40]. As well, existing intervention development frameworks have been criticized for their lack of coherence, comprehensiveness (in terms of not offering the full range of intervention functions to change behavior), and grounding in a model of behavior change [39]. Therefore, this study applied a new framework, the *Behavior Change Wheel* (BCW) framework [14], underpinned by a new model of behavior change, the capability opportunity motivation behavior (COM-B) model [39], designed to incorporate existing theories of behavior change. The BCW incorporates a full range of intervention functions such as education, persuasion, and training that are likely to be effective in eliciting change in a specific target behavior. These intervention functions can be delineated into behavior change techniques (BCTs) using the BCT taxonomy: BCTT (V1) [41], which provides an extensive list of evidence-based BCTs.

The COM-B model defines behavior as part of a system where the three following psychological domains interact to enable a behavior to occur (1) capability (psychological and/or physical; eg, knowledge and skills), (2) motivation (reflective and/or automatic; eg, self-efficacy and emotion), and (3) opportunity (physical and/or social; eg environmental resources and social influences). The model helps to identify which components need to change in order for the target behavior to occur, thus supporting the design of behavior change interventions [40]. Within the BCW, the COM-B model can be further elaborated using the Theoretical Domains Framework (TDF), comprised of 14 theoretical domains drawn from a synthesis of 33 psychological theories and 128 key theoretical constructs relevant for behavior change [42].

Despite the major push to harness mobile phone features that support health behavior change, precisely how to develop theory-informed mHealth interventions that engage users remains a challenge and is rarely well documented in the literature. Therefore, this study provides a detailed outline of how the BCW has been applied in practice for the development of a theory and evidence-driven health promotion app within the context of childhood weight management, whilst also ensuring social validity and engagement amongst the target population.

## Methods

### Overview

**Figure 1:**
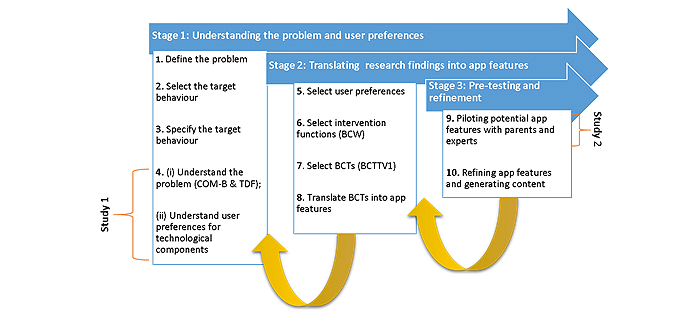
mHealth app intervention development process.

The mHealth app intervention development process followed these three stages (1) understanding the problem and user preferences, (2) translating research findings into app features, and (3) pre-testing the app features for further refinement (Figure 1).

Two empirical studies were conducted at stage 1 and stage 3 of the intervention development process. An iterative feedback loop approach was followed, wherein findings from each stage fed into the next stage of development and were also fed back to refine previous stages.

### Stage 1: Understanding the Problem and User Preferences

#### Overview

The first stage comprised of 4 steps involved in defining the public health problem through to formative research, first with case workers and then parents, on the theoretical, user-centered, and technological components that should be considered in mHealth intervention development.

#### Step 1: Defining the Problem

Step 1 entailed defining the overall health problem in behavioral terms, taking the specific context into account. Hence, an extensive review of the evidence helped to make decisions on whether to focus on children’s eating or exercise behaviors. Based upon this decision, all individuals, groups, and populations potentially contributing to this behavior were considered.

#### Step 2: Selecting the Target Behavior

The first activity at step 2 required a consideration of all the specific behaviors to potentially target in the intervention before narrowing these down to one or two. The BCW recommends a "less is more" approach whereby it is beneficial to start with small changes and build upon these incrementally [14]. Furthermore, discussions with the family weight management services commissioner indicated that the app is one element in a whole range of activities offered as part of the local weight management services. Therefore, it was not necessary to incorporate all possible weight related behaviors into the intervention.

Selecting the target behavior involved conducting empirical research with the target population (refer to step 4), along with consultations with a pediatrician, dietician, and two public health experts in the area of childhood weight management. Hence, focus groups with parents helped to identify the target behavior as well as explore the barriers and facilitators to parents’ capability, opportunity, and motivation for enacting this behavior.

#### Step 3: Specify the Target Behavior

Upon selection of the target behavior, this step involved specifying the behavior and the context in which it occurs (eg, in the supermarket or at home).

#### Step 4: Understanding the Target Behavior and User Preferences

Step 4 involved conducting the first empirical study using a qualitative research design so that both the BCW and UCD methodologies could be simultaneously applied to app development. The former helped to explore barriers and facilitators to parents’ capability, opportunity, and motivation in enacting the target behavior; whereas the latter helped to explore parents’ preferences for app features. The qualitative research involved conducting 6 focus groups, one with family weight management case workers and five with parents.

An intervention mapping table produced from the behavioral diagnosis conducted in the first part of this step served as the basis for mapping theoretical components to app features. The table was reviewed by two health psychologists familiar with the BCW to ensure that the COM-B and TDF theoretical tools had been appopriately applied to the data.

### Data and Sampling (Study 1)

An initial stakeholder meeting with the local public health family weight management commissioning team led to participant recruitment using a purposive sampling strategy. Emails were sent to case workers who were eligible to participate if they had been working with families with overweight children. Parents with overweight and very overweight children were recruited with the help of managers from two local weight management programs. Additionally, parents with healthy weight children were recruited via the university. Parents were eligible if they had a child of ≥5 years and owned a mobile phone.

A total of 48 participants were eligible to take part in this study. Of these, 5 were case workers and the remaining participants were parents. Among the case workers, 4 participated in the study. Of the 43 parents contacted, 22 agreed to participate, yielding a response rate of 51% (22/43) for this group. A total of 6 focus groups were then conducted; 1 with local case workers (4 participants), 4 with parents of overweight and very overweight children (3-4 participants), and 1 with parents of healthy weight children (8 participants). The parent sample comprised predominantly mothers (82%, 18/22), compared to fathers (18%, 4/22). With regards to mobile phone ownership, 77% (17/22) of the sample reported owning a mobile phone. Of those, 41% (7/17) were Android users, 29% (5/17) iPhone users, 18% (3/17) Blackberry users, and 12% (2/17) Windows users. Participants (n=15) recruited from the weight management program had children classified as very overweight (53%, 8/15), overweight (40%, 6/15), and healthy weight (7%, 1/15).

Focus groups were conducted using semi-structured questions developed from a review of existing evidence and structured around the COM-B model and TDF to explore barriers and facilitators to parents’ capability, opportunity, and motivation to provide appropriate food portions for their children (see Textbox 1 for schedule of topics). Additional topics were explored with case workers to gain a deeper understanding of the context of childhood overweight. Topics also revolved around parents’ preferences for healthy eating app features, representing the formative stage of the UCD approach. With the permission of participants, the focus groups were audio recorded and transcribed verbatim.

Transcripts were analyzed by two independent researchers using established principles for conducting thematic analysis [43]. This involved coding segments of data for their basic meaning and mapping these to the COM-B and TDF, as well as coding extracts that referred to users’ preferences for healthy eating app features. An analysis of the data helped to identify which theoretical domains needed to change in order for the target behavior to occur. The BCW refers to this process as the behavioral diagnosis [14].

**Schedule of topics explored with case workers (topics 1-14) and parents (topics 6-14) for study 1.**

Topic

1. Parent's recognition of children's overweight status.
2. Mothers' and fathers' roles in child feeding.
3. Parents' weight status.
4. Barriers to attending family weight management programs.
5. Issues that parents ask for help with.
6. Parents' knowledge of healthful foods and age appropriate portion sizes.
7. Parents' monitoring of children's eating habits.
8. Parents' interpersonal skills around healthy eating and weight issues.
9. Parents' beliefs about consequences of childhood overweight.
10. Parents' beliefs about capabilities of changing children's dietary habits.
11. Parents' emotions (limiting food, talking to children about weight, stress).
12. Other people in parents' environment (other people that make it difficult for parents to provide appropriate food portions).
13. Parents' use of existing technology (websites and apps).
14. Parents' preferences for app features.

### Stage 2: Translating Research Findings Into App Features

#### Overview

Stage 2 was comprised of 3 steps including the selection of user preferences, intervention functions, and behavior change techniques. Collaboration with the commercial app company assisted in operationalising these components into app features.

#### Step 5: Select User Preferences

To help balance the theoretical findings with user preferences, consideration of whether to "reject" or "accept" each user preference was guided using the following criteria (1) relevance to the target behavior, (2) availability online, (3) ease of implementation, (4) alignment with usability and user experience recommendations, and (5) supported from theoretical findings and/or evidence. Consultations with the app company provided insights on the feasibility of user preferences with regards to their implementation (ie, development time and cost).

#### Step 6: Select Intervention Functions

Based on the results of the behavioral diagnosis conducted in step 4, the BCW guided designers on which types of interventions are likely to bring about change in these COM-B components and associated TDF domains. The 9 intervention functions to select from are education, persuasion, incentivisation, coercion, training, restriction, environmental restructuring, modeling, and enablement [14]. The selected intervention functions were then mapped to the intervention mapping table generated from the previous stage.

#### Step 7: Select Behavior Change Techniques

A behavior change technique (BCT) refers to an "active ingredient" and mechanism of change that is an observable, replicable, and irreducible component of a behavior change intervention [44]. Hence, the next step involved delineating intervention functions into BCTs whereupon a candidate list of BCTs was derived from the BCW, linking intervention functions with relevant BCTs [14]. A review of evidence on effective techniques for childhood weight management interventions allowed selection of potentially effective BCTs for use in the intervention such as goal setting [45-47], self-monitoring of behavior [45], and instruction on how to perform the behavior [48]. These were then mapped to the intervention mapping table and reviewed by two health psychologists for further verification.

#### Step 8: Translate Behavior Change Techniques Into App Features

Upon identification of BCTs, steps were taken to embed these as potential app features informed by the user preferences data retrieved from step 4. This involved liaising with the app company with regards to how BCTs could be implemented in the app. With respect to enhancing user experience and engagement, parents’ preferences for app features were built on in consultations with the app company where elements of gamification, defined as the use of game design elements in non-game contexts [49], were applied. For example, progress bars, achievement badges, and points were identified as a way of providing feedback for parents on their children’s eating behavior. In addition, consultations with a software engineer also helped to develop a flow chart of the user journey where BCTs and gamification techniques were linked to specific app features.

### Stage 3: Pre-Testing and Refinement (Study 2)

#### Step 9: Piloting Potential App Features

This step encompassed the second empirical study and involved piloting the proposed features of the app, and seeking feedback for further refinement of app features. Focus groups (n=3) were conducted using similar recruitment strategies followed for study 1. A total of 21 parents were contacted to take part in this study of which 20 took part in the pre-testing phase, yielding a response rate of 95% (20/21). Of the focus groups, 2 were recruited from local weight management programs (7 and 8 participants), consisting of mothers (87%, 13/15) and fathers (13%, 2/15). The third focus group recruited from the university (5 participants) consisted of mothers (60%, 3/5) and fathers (40%, 2/5). Of the participants, three quarters (75%, 15/20) reported owning a mobile phone. Of those, 53% (8/15) owned an Android, 27% (4/15) an iPhone, 13% (2/15) a Blackberry, and 7% (1/15) a Windows device. This study focused on parents’ overall impressions of the app using interactive mock-ups. Focus group discussions were conducted using semi-structured, open-ended questions based on a schedule of topics presented in Textbox 2. The questions drew on a model of usability and user experience goals [50].

A laptop, tablet, and projector were used to present the interactive mock-up. The content of the interactive mock-up was refined after feedback from the first 2 focus groups and an updated version was presented to the last focus group. A thematic analysis similar to the process followed in study 1 was conducted on the data. The salient information from the analysis was then extracted and shared with the app company to help make further iterations to the app’s functional specification.

Lastly, a dietary steering board comprised of two public health dieticians and a family weight management program manager was convened to provide support and feedback on the nutritional content of the app.

**Schedule of topics for study 2.**

Topic

1. Overall impressions of the app
2. Overall helpfulness of app and app features
3. Whether it is perceived as fun and enjoyable
4. Whether it is perceived as satisfying (any features that are liked or not liked)
5. Whether it is perceived as entertaining (mainly referred to content in the quiz feature)
6. Whether certain features are perceived as motivating or not

#### Step 10: Refining App Features, Generating Content, and Developing the Prototype

The final stage involved refining app features based on feedback from parents and the dietary steering board, generating content, and development of the prototype app. The intervention mapping table was completed at this step, where both user preferences and final app features were mapped to relevant theoretical components. Text was used as a mode of delivery for several BCTs in the intervention in the form of two app features: within app text notifications (delivering tips and persuasive messages) and an interactive quiz.

The generation of text for the notifications required a review of the empirical evidence to guide the process of message framing which refers to whether health messages provide benefits of carrying out a behavior (gain-frame) or the consequences of not carrying out the behavior (loss-frame). Generally, gain-framed messages are shown to be more effective for preventative health behaviors, therefore, this framing was used to guide the persuasive messages, tips, and quiz questions in the app [51].

## Results

### Stage 1: Understanding the Problem

#### Step 1: Define the Problem

**Figure 2:**
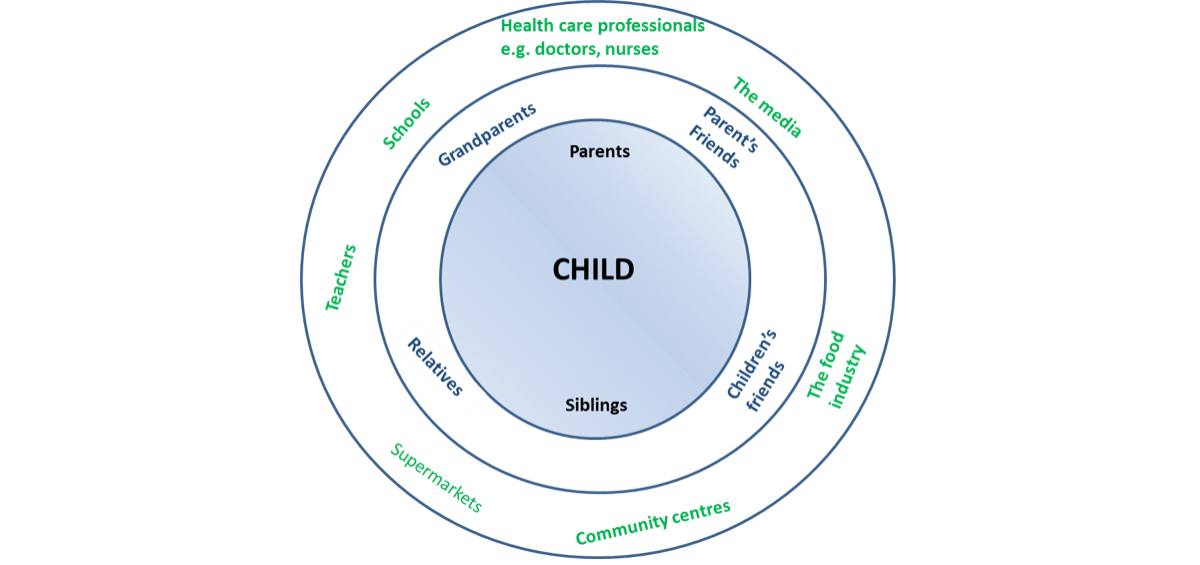
Relevant individuals, groups and populations involved in children's energy intake.

Childhood overweight is a serious public health problem. In England, a third of 10-11 year olds and over a fifth of 4-5 year olds are reported as either overweight or obese [52]. A review of the evidence regarding the subsequent determinants of the energy balance equation provided greater support for focusing on improving children’s diets with regards to reducing their overall energy intake [53-58]. Simultaneously, stakeholder meetings also identified a shortage of online resources in the local area targeting parents to help them improve their family's diets. Consideration of all the individuals, groups, populations, and sectors potentially contributing to children’s energy intake are shown in Figure 2.

Evidence also highlighted the role of parents in children’s energy intake and strongly supported their involvement in childhood weight management interventions [9,10,27,28], including direct involvement in the intervention development process [9]. Hence, this led to the decision to focus on parents as the main population to target in the intervention.

#### Step 2: Select the Target Behavior

Based on the decision to focus on reducing children’s overall energy intake, a range of nutrition behaviors relating to achieving this overall behavior were considered including increasing intake of fruit and vegetables, and reducing intake of saturated fat, sugar, unhealthy snacks, portion sizes, and some carbohydrates. Next, focus groups with parents of overweight children helped to narrow down this list where the problem of children’s consumption of large portion sizes was highlighted as an important behavior to target in the intervention, and one that appeared to be acceptable for parents in changing. Discussions with experts helped to confirm the decision to focus on supporting parents in providing appropriate portion sizes for their children.

#### Step 3: Specify the Target Behavior

Upon selecting the target population, behavior, and setting, the behavior was specified with regards to what needs to occur in order for the target behavior to be carried out (Textbox 3).

**How to specify the target behavior (adapted from [14]).**

Specifications and details

- Target behavior
  - Parents providing appropriate portion sizes (and frequency of food) for their children across the five food groups: fruit and vegetables, protein, dairy, starchy foods, food, and drinks high in sugar and fat (as per the eatwell plate)
- Who needs to perform the behavior?
  - Parents
- What do they need to do differently to achieve the desired change?
  - Preparation, provision of portions (age appropriate portion sizes), and monitoring of food portions
- When do they need to do it?
  - At meal times and snack times
- Where do they need to do it?
  - At home
- How often do they need to do it?
  - Everyday
- With whom do they need to do it?
  - At home
- In what context do they need to do it?
  - The home environment

#### Step 4: Understanding the Target Behavoiur and User Preferences

##### Theoretical Analysis

The behavioral diagnosis revealed barriers to the target behavior (ie, parents providing appropriate portion sizes for their children) in all 3 COM-B components and 10 out of the 14 TDF domains. A full table of the results from the behavioral diagnosis supported with quotes from focus group participants is presented in Multimedia Appendix 1. This information laid the foundations for the beginning of an intervention mapping table used to link each of the components (eg, theory-user preferences in app features) in intervention development.

##### User Preferences for Healthy Eating App Features

Data collected in relation to parents’ ideas and preferences for healthy eating app features formed the first stage of the UCD approach (an excerpt of the results is presented in Table 1). The following 4 key themes emerged from the analysis (1) parents’ preferences for app features (eg, parents preference for the output of recipes from data input on household ingredients), (2) time saving and convenience (eg, parents specified that the app should be simple and quick to use), (3) aesthetics (eg, parents preference for visuals of food), and (4) gamification (eg parents preference for a point system for healthy eating behavior).

**Table 1:** Excerpt of parents preferences for app features.

Themes	Sub-themes	Quotes
App feature	Recipes of household ingredients	I would love to have an app on my phone that says, I have this food what can I do with it… (Parent, focus group 2)
Time saving and convenience	Simple to use	It would have to be quite simple I think. If it got too complicated I just wouldn’t use it. (Parent, focus group 5)
Aesthetics	Visual aids for portion sizes	Pictures were given in the group of the portion sizes... we try to visualize that on the plate so we get roughly the amounts right…I think that would help (Parent, focus group 3)
Gamification	Points for healthy eating	Or you could have something that you could add, what have you had today? Yes I have had one of those, one of those right you get 50 points but I’ve also had one of those, deduct 20 points (Parent, focus group 5)

### Stage 2: Translating Research Findings Into App Features

#### Step 5: Select User Preferences

Focus group discussions resulted in a total of 19 user preferences, wherein 3 were rejected, 5 were partly accepted and 11 were accepted. An excerpt of the user preferences along with reasons guiding decision making (using one or more of the criteria) is shown in Table 2.

#### Step 6: Select Intervention Functions

A total of 6 out of the 9 possible intervention functions were selected: education, training, persuasion, environmental restructuring, enablement, and modeling (parent/child). Table 3 shows an excerpt of how these intervention functions were mapped to the corresponding COM-B and TDF components, along with examples of how they could be applied to supporting parents’ portion control behaviors with their children.

**Table 2:** Excerpt of decisions for rejecting or accepting user preferences.

Main theme	User preference	Accept /reject	Reason
**App features**			
	Every family is different so they need to be able to choose their own goals	Accept	(v) Supported by literature (+)^a^
	Recipe output of household ingredients	Reject	(i) Aligned with target behavior (-)^b^; (ii) already apps and websites that provide this (-); (iii) not within budget (-)
**Usability**			
	Needs to be minimal data input	Accept	(iv) Aligned with recommended usability goals (+); (v) parents lack of time was identified as an important barrier to make changes (+)
**Aesthetics**			
	Visuals of food in the app	Accept	(iv) Aligned with recommended user experience goals (+)

^a^(+) Aligned with criterion

^b^(-) Not aligned with criterion

**Table 3:** Excerpt of mapping intervention functions to COM-B and Theoretical Domains Framework (TDF) components.

COM-B	Theoretical Domains Framework (TDF)	Sub-themes	Intervention funtions	Example
Psychological capability	Skills (cognitive)	Parents have difficulty in measuring food portions	Training, Environmental restructuring	Train parents to measure portion sizes, provide a visual tool to help measure food
Reflective motivation	Beliefs about capabilities	Parents have a lack of confidence in their ability to make changes	Education, persuasion, Enablement	Educate, persuade and enable parents to increase their self-confidence in making changes to their children’s eating habits.
Physical opportunity	Environmental context and resources	Parents’ preferences for household objects such as plates to measure portion sizes	Environmental restructuring	Restructure the home environment to provide a tool for greater accuracy in measuring food portions

#### Step 7: Select Behavior Change Techniques

A total of 21 BCTs were selected at this step and mapped onto the intervention functions, TDF, and COM-B components as shown in Table 4.

#### Step 8: Translate Behavior Change Techniques Into App Features

Consulting with the app company facilitated the process of how BCTs identified in step 7 could be meaningfully combined with findings on user preferences (step 4) to create app features. Table 5 provides an example of this process where each BCT is mapped to each user preference and proposed app feature.

Further elements of gamification techniques, shown in Textbox 4, were recommended by the app company to increase parents’ motivation in completing tasks, such as logging food and answering quiz questions. These also related to specific BCTs.

**Figure 3:**
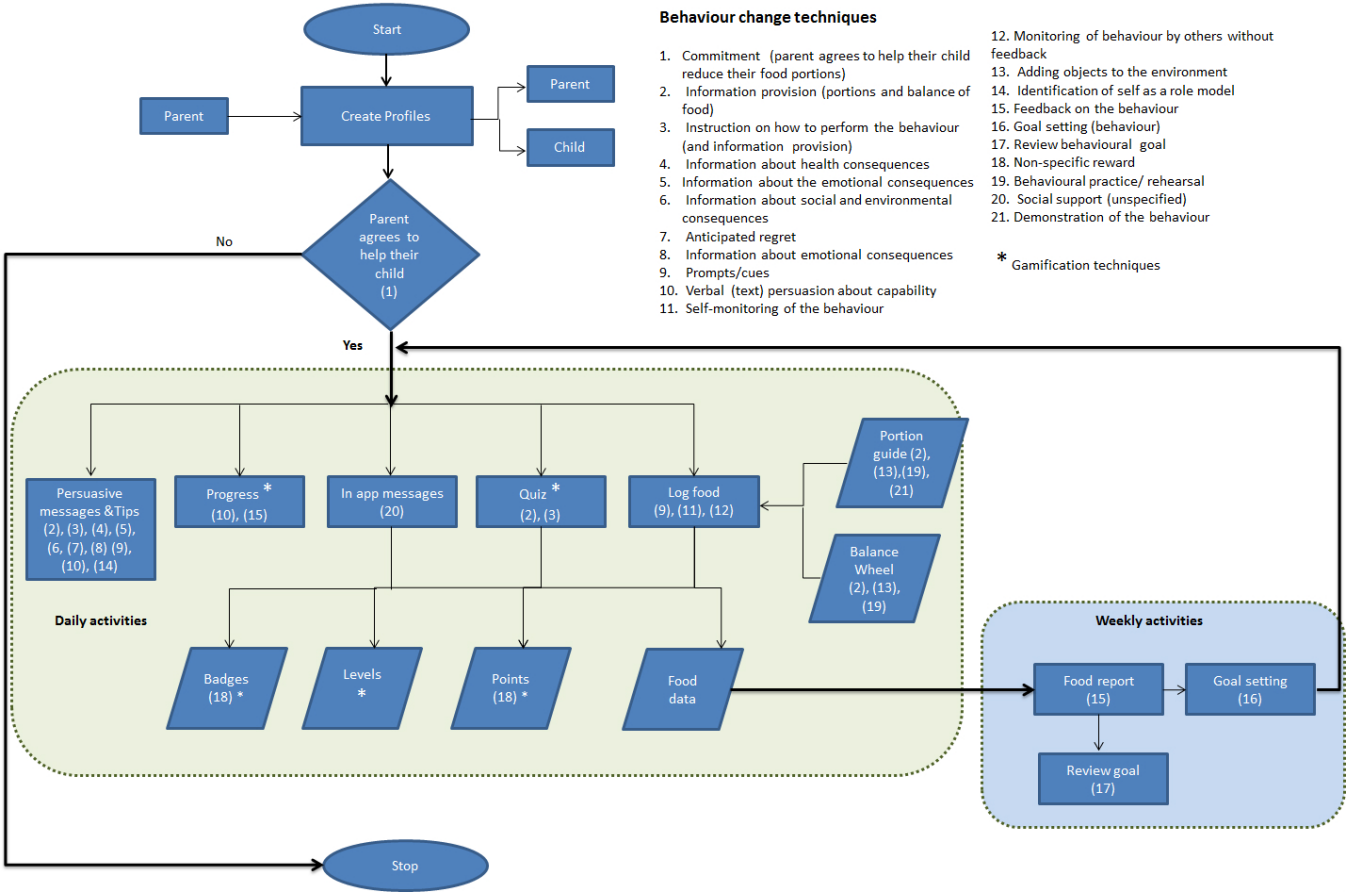
Intervention flow chart.

Consultations with a software engineer led to the development of a flow chart (Figure 3) to help map specific BCTs to app features, showing the sequence of intervention components delivered to parents. The diagram was further refined through parental feedback in the next stage of development.

**Figure 4:**
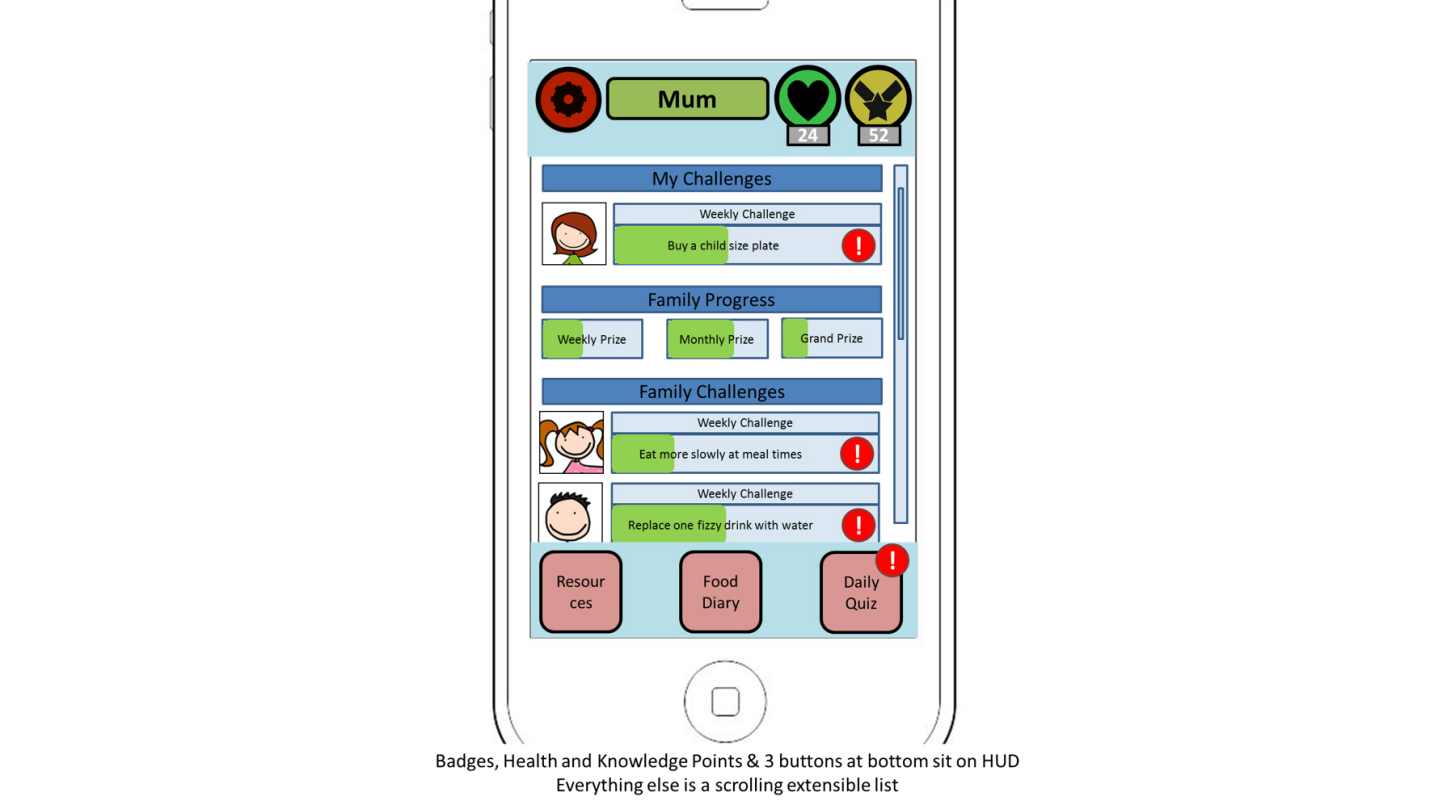
Interactive mock-up of homescreen.

Additionally, interactive mock-ups of the app were developed by the app company and used to pilot the proposed features with parents. An example of the home screen is shown in Figure 4.

**Table 4:** Mapping behavioral change techniques (BCTs) to intervention functions.

COM-B	Theoretical Domains Framework (TDF)	Intervention funtions	Behavioral change techniques (BCTs)
**Psychological capability**			
	Knowledge	Education	Instruction on how to perform the behavior, habit formation
	Memory, attention, and decision making skills	Training	Instruction on how to perform the behavior, behavioral practice/rehearsal/, habit formation
	Skills (cognitive and interpersonal)	Training, enablement	Instruction on how to perform the behavior, behavioral practice/rehearsal/, habit formation
	Behavioral regulation	Training, enablement, modelling	Monitoring of behavior by others without feedback, self-monitoring of behavior, feedback on behavior
**Reflective motivation**			
	Intentions	Persuasion	Commitment
	Social identity	Persuasion, modelling	Identification of self as role model, valued self-identity
	Beliefs about capabilities	Persuasion, training	Instruction on how to perform the behavior, goal setting, feedback on behavior, prompts/cues
	Beliefs about consequences	Education, persuasion, Training	Information about health consequences, information about social and environmental consequences
**Automatic Motivation**			
	Emotion	Persuasion	Social support (emotional), self-monitoring of behavior
**Physical opportunity**			
	Environmental context and resources	Environmental restructuring	Adding objects to the environment, restructuring the physical environment
**Social opportunity**			
	Social influences	Enablement	Social support (unspecified), social support (practical)

**Table 5:** Excerpt of examples of behavioral change technique (BCT) user-centered design (UCD) app translation.

Behavioral change techniques (BCTs)	User preferences	App features
Instruction on how to perform the behavior	Time saving and convenience, visual aids	Balance wheel, portion guide tool
Self-monitoring of the behavior	Gamification	Points for logging food
Feedback on the behavior	Time saving and convenience, visual aids,	Visual feedback of food groups to target in the following week
Non-specific reward	Gamification	Points and awards for completing activities

**Gamification techniques and behavioral change techniques (BCTs).**

Technique

- Points
  - Non-specific reward
  - Feedback on the behavior
- Achievements
  - Non-specific reward
  - Feedback on the behavior
- Progress bars
  - Feedback on the behavior
- Quiz
  - Information provision
  - Instruction on how to perform the behavior
  - Information about health consequences

### Stage 3: Pre-Testing and Refinement of App Features

#### Overview

This stage involved the final 2 steps of the intervention development process where proposed app features were piloted with parents, content was generated and refined through consultations with experts, and the prototype app developed.

#### Step 9: Piloting Potential App Features

Feedback on the interactive mock-ups provided valuable insight into parents’ impressions of the proposed app content. For example, the ideas identified around gamification were also piloted with parents to ensure that they were in line with parents’ interests [59]. Hence, this stage focused on usability and user experience components such as whether the app features were perceived as fun, helpful, motivating, and aesthetically pleasing. This information was extracted and organized into the following three themes of feedback (1) app features (eg, parental feedback on the portion guide tool), (2) gamification (eg, parents feedback on gamification features), and (3) app positioning (eg, parents feedback on the app positioned as a healthy eating app as oppose to a weight management app) (Table 6).

**Table 6:** Excerpt of results from pilot testing interactive mock-ups with parents.

Theme	Sub-theme	U^a^ codes	UX^b^ codes	Example quote
App feature	Portion guide	Easy to remember how to use (+)^c^, easy to learn (+),	Satisfying (+), helpful (+)	I think the bit with the hands and the portion sizes, I think that’s really really good as it’s so difficult to know what a portion size is and very easy to use (Parent, focus group 9)
Gamification	Competition against other families	Safe to use (-)^d^	Motivating (-)	Not too sure about that one as my son has a real complex about his weight so I think it would be tough on him to see other people and might get ‘oh well they are doing better than me’, do you know what I mean? It’s like a confidence thing (Parent, focus group 8)
App positioning	Healthy eating app		Emotionally fulfilling (+), satisfying (+)	I like the idea that it’s about healthy eating, you know, not weight control, I like the name as well (Parent, focus group 9)

^a^U: usability

^b^UX: user-experience

^c^Positively viewed (+)

^d^Negatively viewed (-)

#### Step 10 - Refining App Features, Generating Content and Developing the App Prototype

Following the aforementioned steps led to the development of a prototype app where the theoretical and user-centered components were systematically linked to app features. The overall concept of the app, final intervention mapping table, and examples of text content are presented below.

### Overall Concept of the App

Once parents have downloaded the app onto their mobile phone and set up user profiles for family members, they must agree to help their children reduce their food portions before they can participate. All users are instructed to log their food using the camera function, indicating which food group they have eaten and how many portions, using the balance wheel and portion guide as references. The quiz feature offers users a new quiz question every day in relation to portion sizes and the balance of food groups. Once users have logged their food for one week, they will receive a visual report of their food portion intake, highlighting the food groups users may like to set goals in reducing portions in. Users can compose messages to send to other family members requesting help in achieving their weekly goals. Users will receive points for answering quiz questions, logging their food, and helping other family members. Feedback on users’ progress towards their weekly goal will be shown visually in progress bars. Parents will receive daily notifications, within app text messages and feedback with regards to their child’s progress towards their weekly goal. Parents are also signposted to local family dietician services, group weight management programs, and healthy recipes (see Multimedia Appendix 2).

### The Final Intervention Mapping Table

The results of the final mapping table where each theoretical, user-centered, and app feature have been linked together can be viewed in Multimedia Appendix 3. It is important to consider that once app features have been developed, they may incorporate other BCTs that were not originally part of the mapping process which has been documented by other researchers in the field [60].

### Content

Table 7 shows an example of the content that was generated for the persuasive messages (via within app messages and notifications), tips, and quizzes.

**Table 7:** Excerpt of content for within app messages, notifications, and quiz questions.

COM-B	Theoretical Domain Framework (TDF)	Behavioral change techniques (BCTs)	Example
Motivation	Beliefs about capabilities, Beliefs about consequences	Feedback on the behavior, Information about health consequences	Well done! By helping your child to maintain a healthy weight you will reduce their risk of becoming an overweight adult^a^.
Capability	Knowledge	Instruction on how to perform the behavior	One glass of apple juice and one glass of orange juice count as how many portions of fruit^b^?

^a^Example of content for notifications and messages on loading screens

^b^Example of text content for quiz

## Discussion

### Principal Findings

Mobile phones possess a range of attributes that can facilitate health promotion apps to support behavior change; however, the development of a majority of these apps available to consumers has occurred in isolation of theory and evidence, resting mainly on developers’ intuitions [60]. To date, few published research studies have provided enough detailed information about the steps involved in the development of a mHealth app that can be replicated [21]. This paper provides a step by step exemplar for how evidence, theory, and user-centered components were incorporated into a mHealth app.

A behavioral diagnosis using the BCW revealed that parents experienced barriers in their capability, opportunity, and motivation to provide appropriate portion sizes for their children. This led to the selection of 6 intervention functions and 21 behavior change techniques to bring about change in this target behavior. Findings with regards to parents’ preferences for app features revolved around their desires for specific app features such as a recipe tool, simple and quick interactions with the app, visual aids, and elements of gamification such as scoring points for healthy eating. Techniques of gamification were further expanded to increase parents’ engagement with the app and deliver specific BCTs.

A major strength of this study was the involvement of multiple stakeholders in the app development process including the local authority, family weight management service commissioners, community program managers (for recruitment of parents with overweight children and the implementation of the app), family case workers, parents of overweight and healthy weight children (for ensuring social validity amongst the target population), pediatricians, dieticians, psychologists (for the nutritional and psychological content), mobile app experts, and software engineers (for the translation of research findings into app features and technical development of the app). Hence, results can serve as a systematic framework for developers in terms of incorporating stakeholder-informed design elements in the development of health promotion apps.

An additional strength was the use of a comprehensive framework (BCW) where one of the major components differentiating it from other behavior change intervention frameworks is that it is underpinned by a model of behavior change. The COM-B model embodies a 360 degree comprehensive inclusion of behavior change theories. It is a dynamic model that, unlike other theories of behavior change, takes account of the context of behavior, automatic processes (eg, habit, emotion), and environmental influences [61,62]. Furthermore, the BCW uses a standardized language of theoretical constructs and behavior change techniques which is essential for the replication and synthesis of research and evidence [63]. However, similar to other psychological models and health behavior change intervention frameworks, the BCW stops short of serving as a guide when it comes to translating behavior change techniques into mHealth app features due to the infancy of the mHealth field. Additionally, it’s execution relied heavily on the expertise, creativity, and judicious decision making of the design team with regards to which components should actually be implemented in the app, as well as drawing on existing evidence, practical considerations, end-users’ views, and expert advice. Thus, it is necessary to expand on the BCW using other disciplines in design and engineering and collaborate with the commercial app industry for the development of behavior change interventions that is relevant for the mobile app ecosystem. Fundamentally, the UCD approach yielded granular information on the relevance, acceptability, and preference of app features within the target group. Consultations with the app company combined with the research findings allowed for the application of *meaningful gamification* which can help parents to manage their children’s eating habits as well as promote engagement [59].

Since conducting this study, a new framework, Behavioral Intervention Technology (BIT) model, has been published which attempts to integrate both conceptual and technological components of electronic health (eHealth) and mHealth interventions [60]. In particular, it offers a method for targeting distal clinical aims (eg, weight reduction) and translating behavior change strategies into an app features. However, in contrast, the BCW approach starts with a behavioral diagnosis of the target behavior, before moving onto the possible solutions. Furthermore, the BIT model does not integrate UCD elements, a crucial step that was followed in our work in ensuring the social validity of the app amongst the target population.

### Limitations

There are several limitations with the current approach that must be acknowledged. Firstly, the time taken to develop a prototype app is an important consideration, a factor which has also been reported for other mHealth interventions [21,64]. Compared to intervention development, the associated technology development occurs at a much faster pace [65]. Consequently, by the time mHealth interventions are implemented and tested, the technology may have potentially moved on. Secondly, systematically developing a health promotion app intervention can also be resource intensive. In this study, incorporating UCD revealed the need for further refinement of app features which is a much needed step. At the same time, implementing the changes requires additional resources.

With regards to the specific target behavior, this paper focused on both actual portion sizes and frequencies of portions. Nevertheless, there are other nutrition behaviors that can be targeted in the app, along with exercise behaviors, which provides opportunities for further research. One solution may be to target behaviors at different periods of time. For example, after families have completed a 12-week intervention targeting portion control, they could then have the opportunity to move onto the next stage where a new behavior is targeted.

The empirical research used a qualitative study design, whereas quantitative surveys have typically been applied to research designs seeking the most appropriate targets for interventions to date [66,67]. However, the BCW approach does not require this, partly because the COM-B model includes factors that go beyond the sociocognitive spectrum (eg, opportunity) and questionnaires can only measure perception of opportunity rather than objectively assess this.

### Future Research

In addition to applying techniques of gamification to the intervention as a way to help increase parents and families’ engagement with the app, the study highlighted other important components that have the potential to increase (and decrease) parents’ engagement with the app such as interactivity, novelty, and tailoring of app content. Engagement is a multidimensional construct; hence further research with the prototype app drawing on a validated model of user engagement [68] will be necessary. This would provide insight into which aspects are important for capturing parents’ attention and encouraging their sustained use of the app.

The next stage in development will involve formal usability testing with parents which will result in further refinements prior to conducting an evaluation of the impact of the app on families’ portion sizes. Lastly, the app is developed specifically for a parent population with young children. We encourage researchers to apply the developed methodology to other samples as this can help to refine and expand on the app intervention development process

### Conclusions

Within the context of mHealth interventions, we cannot ignore the reality that theoretical, user-centered, and technological components are inexorably linked. Simultaneous consideration must therefore be afforded to them, following a systematic development process that draws on relevant theory, evidence, and research with the target population. In this paper it has been demonstrated how the BCW can serve as a systematic and comprehensive guide to ensure that a health promotion app is underpinned with relevant theory and evidence. Integrating this step by step approach with activities and methods from user-centered design and collaboration with the commercial app industry has also been clearly explicated. This work provides a template and practical guide for researchers and app developers looking to apply similarly systematic and rigorous approaches to content development of mHealth interventions in the future.

## Appendix

Multimedia Appendix 1 COM-B behavioral diagnosis.

Multimedia Appendix 2 Health Heroes screenshots.

Multimedia Appendix 3 Final intervention mapping table.

## Abbreviations

BCT: behavioral change technique
BCW: behavioral change wheel
mHealth: mobile health
PDA: personal digital assistants
TDF: Theoretical Domains Framework
UCD: user-centered design
