# Supplementary material for: Targeting Parents for Childhood Weight Management: Development of a Theory-Driven and User-Centered Healthy Eating App
Source: JMIR Mhealth Uhealth. 2015 Jun 18;3(2):e69. doi: 10.2196/mhealth.3857 (PMC4526951; doi:10.2196/mhealth.3857)
Supplement: Multimedia Appendix 1 [file mhealth_v3i2e69_app1.pdf]

Com-B	TDF	Sub-themes	Example quotes – Change4Life advisors (Focus group 1)	Example quotes - Parents (Focus groups 2-6)
Psychological Capability	**Knowledge, Memory, attention and decision making processes**	*Parents lack knowledge and have a difficulty in making decisions on appropriate portion sizes*	But when I say to people what's your child's portion size in relation to yours..I mean one dad, said 'well actually she eats the same as me' cos they've had a Sunday Dinner and the daughter who is 11 would have the same size meal as him	Until I came here, I didn't really know much about portion sizes at all
		Parents lack knowledge on the balance of food groups	You often don't get the families that understand the balance do they of foods..you know you get the 'eatwell' plate with tiny portions of fats..and..I don't know if everyone went to training last week but the 'naughty but nice'section. (laughs).I just don't think they understand how much one chocolate bar actually is or how much fat is actually in a portion of chips, it's very hard to try and get that across I think..	P3: It's difficult isn't it? I think maybe, well what we've done, the picture were given in the group of the portion sizes of what food groups you should have what quantity of and we've got that on the dining room table and the kids actually say that's me chicken and that's me veg and we try to visualise that on the plate so we get roughly the amounts right, the portion's too big but the ration is roughly right but i think if there was like a poster or a plastic plate that you could get for a 6 year old, an 8 year old and a 10 year old, so that you knew, roughly what you should be giving and i think that would help
	**Skills (ability)**	*Parents' difficulty in measuring food portions*	But when I say to people what's your child's portion size in relation to yours..I mean one dad, said 'well actually she eats the same as me' cos they've had a Sunday Dinner and the daughter who is 11 would have	For me, I find it particularly difficult dishing out the correct portion size for children and for adults, I suppose. I just tend to give everybody the same amount. I suppose I've, sort of, justified that in a way that I'm dieting so I'm actually having less of a portion size, so hers was equal to mine and that's

			the same size meal as him	the way I, sort of, justified it in my mind, but that's probably not the right way to do it
	**Skills (interpersonal)**	*Parents' difficulty in Communicating with their children and partners around eating and weight issues*	I actually think they find it quite difficult, especially at Year 6 when the young people are starting to formulate their own habits and they do have a lot of opportunity to kind of go against what parents are advising as well really, and I think it's quite difficult to get the message across and encourage that health change behaviour without becoming something else that is a bit of a battle	Yeah, I think if you give them a smaller portion size, then Daisy will say why have I got less and doesn't really understand that she's got a smaller body and needs less than adults do.
	**Behavioural regulation**	*Parents' regulation of their own portion sizes and their children*	But when I say to people what's your child's portion size in relation to yours..I mean one dad, said 'well actually she eats the same as me' cos they've had a Sunday Dinner and the daughter who is 11 would have the same size meal as him	From my own experience, you tend to give them what you see as adequate, that's your mind thinking more about you more than the kid, so I know for a fact we give our kids too large portions, far too big, but when we do measure them out and put them in the bowl it doesn't seem much but it's adequate for them so in effect we are over filling them
**Reflective Motivation**	**Social identity**	*The role of the mother*	Even when you phone up they are like 'oh you need to speak to my wife about that' or mother about that..it's like they can't make a	All that takes time to prepare and you don't have that time.

			decision..or they don't want to deal with it	
	**Beliefs about capabilities**	*Parents have a lack of confidence in their ability to make changes to their children's dietary behaviours and weight*	I think if you've got..I mean i've spoken to mum's and they've still tried to lose weight in the past and tried every diet going so they've tried it all for themselves. How will are they to try it for their child?	What are your thoughts on how confident you feel in successfully making changes to your children's diet?<br><br>'I'd give in too easily'<br>'Not very confident'
	**Beliefs about consequences**	*Parents' difficulty in linking childhood and adulthood overweight with health risks*	P3:I've got one family where mom's got diabetes, dad's dad died of a heart attack really young, his brother had died really young, he wasn't in good health at all, mom wasn't in good health. The one day I went for a visit and her sister had just been taken into hospital with a heart attack but they couldn't relate that to any sort of..<br>M: being overweight<br>P3: No	I think for me it's the teasing, you know, the peer pressure because I was teased really badly at school for being overweight and that's my main memory of secondary school just being told you're fat and you know and I don't want them to go through that.

		Parents believe that portion sizes take too long to measure	N/A	I don't weight out my portion to know what it is..or even if they say per so many grams, I still don't have the time to figure out..I just don't want to
		Parents' belief that they do not need to change children's eating behaviour if they do more exercise	..they do quite a lot of exercise and so they feel they're doing really well when actually even the people who do go to football or Rugby three times a week, they're only doing what they should be doing anyway….and they're then giving that food extra food which they don't need	Yeah..we're just trying to get him more active, badminton, squash and cycling, stuff like that. With the weather coming..so we're hoping to exercise it off him (laughs) rather than doing anything too drastic with his diet, we're putting more veg on
**Automatic Motivation**	**Emotions**	*Parents' fear of child becoming anxious about weight/eating disorders*	when you're contacting year 6 parents where the young people are overweight..the parents don't necessarily want to bring up the subject because of they're age.	Well he's only just joined the senior school so I don't want to overload him with too many worries …we don't want him to go the other way and you know..have anxieties about that
		Parents' denial/recognition of the problem	They don't believe they're overweight ..they're just like their brothers and sisters	I thought this was about sport when we first comes here, I didn't realise my son was overweight..I was quite..oh my goodness
		Parents' guilt of restricting food	I think some families its more..I love you to death..cos they don't stop their child from having that chocolate bar and saying to them	I'll just make anything into a guilt trip for myself I think that's all. So food is one of those.

			'you can't have that chocolate bar but you can have a piece of fruit instead'. They haven't got that strength in them because they feel like they are being mean	
Physical Opportunity	**Environmental context and resources**	*Parents' resources for measuring portion sizes*		yeah tea cup and then it would be so much easier..<br><br>And this is what my issue with my husband giving them far too much is that he is using pasta bowls that's adult size deep dish bowls that go one forever..
		Not enough time to figure out portion sizes		I don't weight out my portion to know what it is..or even if they say per so many grams, I still don't have the time to figure out..I just don't want to
Social Opportunity	**Social influences**	Grandparents	..On a positive note..um..some of my families..they are really trying to make this change but Grandma..they go over to Grandma's and Grandma is giving them all this stuff!!	When he was writing his meal for yesterday, bearing in mind that I thought I was with him all day yesterday, because we all live with my parents, and he said 'I had a penguin' and I said 'when did you have that? Did Nanny give it ya?' 'Yeah'.
		Partners		Well I'm relying on the kids now to say to daddy you need to put some vegetables on the plate because I've given up trying to tell him..
