# Supplementary figures and images for: Targeting Parents for Childhood Weight Management: Development of a Theory-Driven and User-Centered Healthy Eating App
Source: JMIR Mhealth Uhealth. 2015 Jun 18;3(2):e69. doi: 10.2196/mhealth.3857 (PMC4526951; doi:10.2196/mhealth.3857)

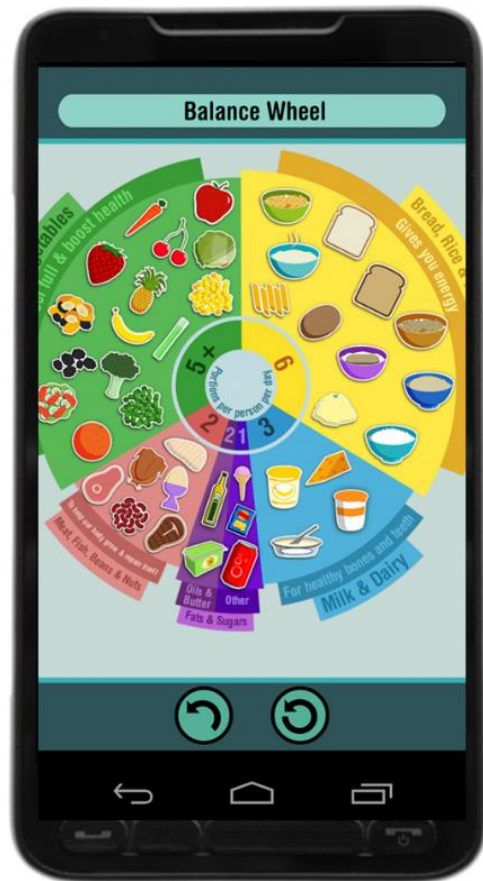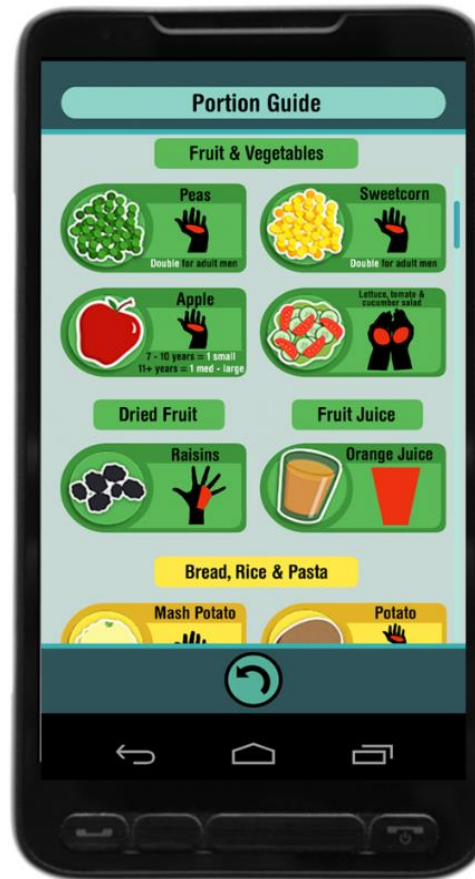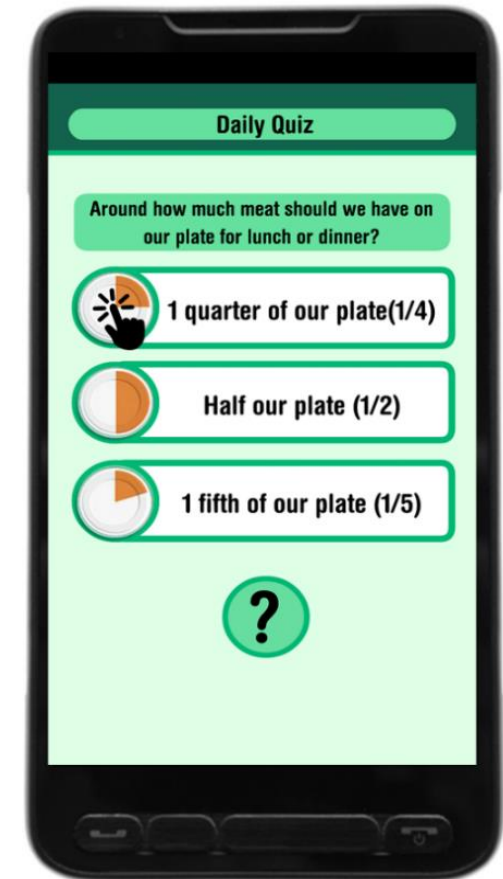

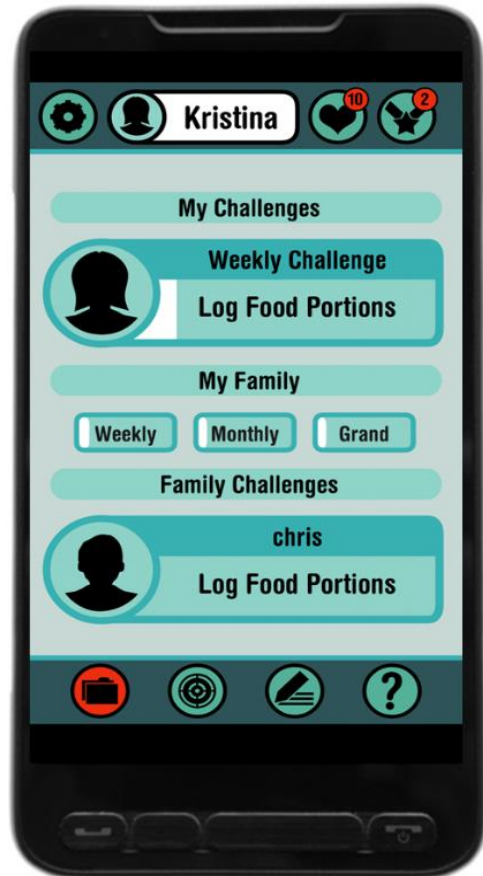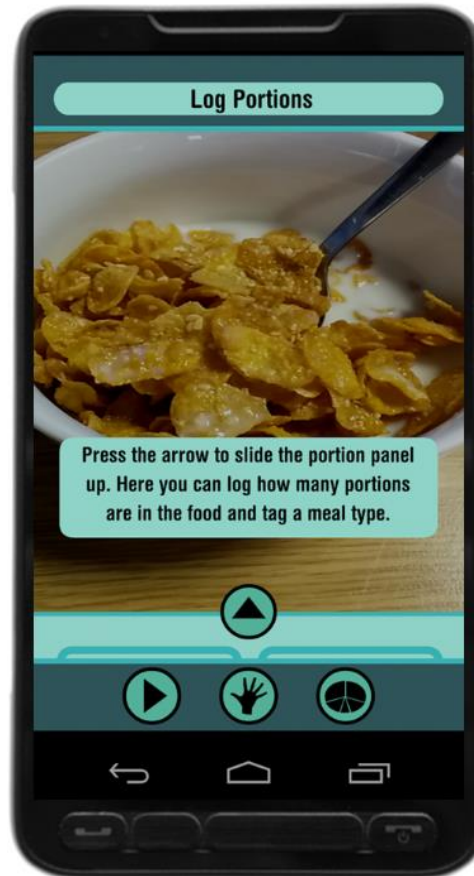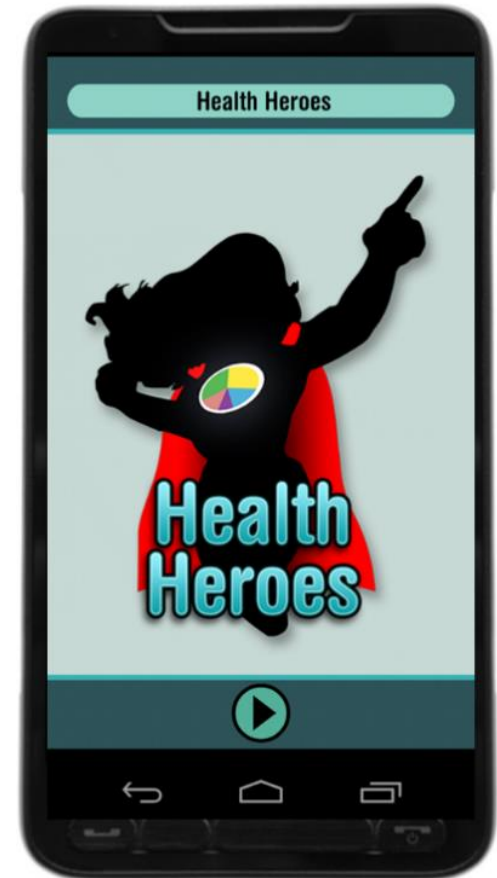

Supplement: Multimedia Appendix 2 [file mhealth_v3i2e69_app2.pdf]
