# Supplementary material for: Targeting Parents for Childhood Weight Management: Development of a Theory-Driven and User-Centered Healthy Eating App
Source: JMIR Mhealth Uhealth. 2015 Jun 18;3(2):e69. doi: 10.2196/mhealth.3857 (PMC4526951; doi:10.2196/mhealth.3857)
Supplement: Multimedia Appendix 3 [file mhealth_v3i2e69_app3.pdf]

Stage 1 (Steps 1-4)			Stage 2 (Steps 5-8)			Stage 3 (Steps 9-10)
**COM-B**	**TDF**	**Sub-themes**	**User preference**	**Intervention Functions**	**BCTs**	**App features**
**Psychological Capability**	**Knowledge, Memory, attention and decision making processes**	Parents lack knowledge and have a difficulty in making decisions on appropriate portion sizes	Time saving and convenience (Quick and simple), visual aids	Education, Training, *Environmental restructuring*	(2) Provide information on appropriate portion sizes (information provision), (3) Instruction on how to perform the behaviour, (15) Feedback on the behaviour, (13) *Adding objects to the environment*	Portion guide tool, tips via notifications and in app messages, progress bars
	**Knowledge, Memory, attention and decision making processes**	Parents lack knowledge on the balance of food groups	Time saving and convenience (Quick and simple), visual aids, Communication (tools they can use together)	Education, Training	(2)Provide information on portion guidelines for food groups (information provision), (3) Instruction on how to perform the behaviour, (15) Feedback on the behaviour	Balance wheel, tips via notifications and in app messages, food reports

	**Skills (Cognitive)**	Parents have a difficulty in measuring in food portions	Time saving and convenience (Quick and simple), visual aids, Communication (tools they can use together)	Training	(3) Instruction on how to perform the behaviour, (19) Behavioural practice	Portion guide tool, tips
	**Skills (interpersonal)**	Parents have a difficulty in Communicating with their children and partners around eating and weight issues	Communication (tools they can use together), Gamification	Enablement	(13) Adding objects to the environment	The app itself is designed to foster communication by being a tool for the family to use together
**Reflective Motivation**	**Behavioural Regulation**	Parent's regulation of their own and their children's portion sizes	Minimal data input, quick and simple, tools to use together, visual aids, Gamification-challenge for the whole family to compete in	Training, Enablement, Modelling	(17) Goal setting of behaviour, (11) Self-monitoring of behaviour, (14) Identification of self as a role model (parental modelling) (21) Demonstration of the behaviour, (18) Non-specific reward	Food logging section, goal setting section, Persuasive messages via notifications and in app messages

	**Intentions**	Parents' decisions to take action	Gamification (A challenge for the family to compete in)	Persuasion	(1) Commitment	Parents must agree to help their children achieve the target behavior before they begin the challenge
	**Social identity**	Role of the Mother	Communication (tools they can use together), Gamification	Persuasion, Modelling	(14) Identification of self as a role model, (10) Verbal (text) persuasion about capability	Persuasive messages via within app text notifications
	**Beliefs about capabilities**	Parents have a lack of confidence in their ability to make changes	Time saving and convenience (Quick and simple), visual aids, Communication (tools they can use together)	Persuasion, Training	(11) Self-monitoring of the behaviour, (17) Goal setting of the behaviour, (15) Feedback on the behaviour, (14) Identification as a role model, (3) instruction on how to perform the behaviour	Persuasive messages via within app text notifications, badges, progress bars, achievements, quiz, nutrition tools
	**Beliefs about consequences (childhood overweight and providing**	Parents' beliefs of the health risks of childhood overweight	N/A	Education, persuasion,	(4) Information about health consequences, (6) Information about social and environmental consequences	Persuasive messages via within app text notifications

	**appropriate portion sizes)**	Parents' belief that portion sizes take too long to measure	Time saving and convenience (Quick and simple),	Education, persuasion, Training	(3) Instruction on how to perform the behaviour, (19) Behavioural practice, (10) Verbal (text) persuasion about capability	Portion guide tool, Persuasive messages via within app text notifications
**Automatic Motivation**	**Emotions**	Fear of child becoming anxious about weight/eating disorders if they talk to them about their dietary behaviour	N/A	Persuasion	(4) Information about health consequences, (6) Information about social and environmental consequences	Persuasive messages via within app text notifications
		Denial/recognition of the problem	N/A	Persuasion	(4) Information about health consequences, (8) Information about emotional consequences	Persuasive messages via within app text notifications
		Parents' guilt of restricting food	Gamification (a challenge the whole family can compete in)	Persuasion	(8) Information about emotional consequences, (7) Anticipated regret	Persuasive messages via within app text notifications

**Physical opportunity**	**Environmental context and resources**	Parents' resources for measuring portion sizes	Time saving and convenience (Quick and simple), visual aids,	Environmental restructuring	(13) Adding objects to the environment, (9) Prompts/cues	The App (is an object added to the environment) Resources section - for measuring food portions
**Social opportunity**	**Social influences**	Grandparents like feeding children more food items	Communication (tools they can use together), Gamification	Enablement	(20) Social support (unspecified)	The app provides a challenge which the whole family can compete in. Users can send requests via within app messages to other family members to help achieve their goals. The family member receives points for agreeing to help. .
	**Social influences**	Partners provide children with large portions	Communication (tools they can use together), Gamification	Enablement	(20) Social support (unspecified)	
